# Atomic-level passivation mechanism of ammonium salts enabling highly efficient perovskite solar cells

**DOI:** 10.1038/s41467-019-10985-5

**Published:** 2019-07-08

**Authors:** Essa A. Alharbi, Ahmed Y. Alyamani, Dominik J. Kubicki, Alexander R. Uhl, Brennan J. Walder, Anwar Q. Alanazi, Jingshan Luo, Andrés Burgos-Caminal, Abdulrahman Albadri, Hamad Albrithen, Mohammad Hayal Alotaibi, Jacques-E. Moser, Shaik M. Zakeeruddin, Fabrizio Giordano, Lyndon Emsley, Michael Grätzel

**Affiliations:** 1 Laboratory of Photonics and Interfaces, Institute of Chemical Sciences and Engineering, School of Basic Sciences, Ecole Polytechnique Fédérale de Lausanne, CH-1015 Lausanne, Switzerland; 2 National Center for Nanotechnology, King Abdulaziz City for Science and Technology, Riyadh, 11442 Saudi Arabia; 3 Laboratory of Magnetic Resonance, Institute of Chemical Sciences and Engineering, School of Basic Sciences, Ecole Polytechnique Fédérale de Lausanne, CH-1015 Lausanne, Switzerland; 4 Photochemical Dynamics Group, Institute of Chemical Sciences and Engineering, Lausanne Centre for Ultrafast Science, École polytechnique fédérale de Lausanne, CH-1015 Lausanne, Switzerland; 5 Physics and Astronomy Department-Research Chair for Tribology, Surface and Interface Sciences, College of Science, and King Abdullah Institute for Nanotechnology-Aramco Laboratory for Applied Sensing Research, King Saud University, Riyadh, 11451 Saudi Arabia; 6 Present Address: Laboratory for Solar Energy and Fuels, The University of British Columbia, Kelowna, BC V1V 1V7 Canada; 7 Present Address: Institute of Photoelectronic Thin Film Devices and Technology, Key Laboratory of Photoelectronic Thin Film Devices and Technology of Tianjin, Nankai University, 300350 Tianjin, China

**Keywords:** Chemistry, Energy science and technology, Materials science

## Abstract

The high conversion efficiency has made metal halide perovskite solar cells a real breakthrough in thin film photovoltaic technology in recent years. Here, we introduce a straightforward strategy to reduce the level of electronic defects present at the interface between the perovskite film and the hole transport layer by treating the perovskite surface with different types of ammonium salts, namely ethylammonium, imidazolium and guanidinium iodide. We use a triple cation perovskite formulation containing primarily formamidinium and small amounts of cesium and methylammonium. We find that this treatment boosts the power conversion efficiency from 20.5% for the control to 22.3%, 22.1%, and 21.0% for the devices treated with ethylammonium, imidazolium and guanidinium iodide, respectively. Best performing devices showed a loss in efficiency of only 5% under full sunlight intensity with maximum power tracking for 550 h. We apply 2D- solid-state NMR to unravel the atomic-level mechanism of this passivation effect.

## Abstract (web summary)

Various approaches have been developed to push higher the efficiency of halide perovskite solar cells. Here Alharbi et al. show that ammonium salts treatment can reduce the defect density at the perovskite surface and understand the passivation mechanism with 2D-solid state NMR.

## Introduction

Metal halides perovskite are one of the most promising light harvesting materials among emerging photovoltaic technologies^1–5^ solar to electric power conversion efficiencies (PCEs) reaching presently 23.7%^6^. Their ease of manufacturing together with low cost fabrication and high performance have made metal halide perovskites a true breakthrough in the thin film solar cell technology. However, solution deposition methods are prone to produce pinholes and defects whether at the grain boundaries or at the surface, which is considered one of the reasons behind a low device performance and stability^7,8^. One major obstacle to the development and commercialization of this technology continues to be the operational stability of the photovoltaic devices. Despite the vast progress that has been achieved on the synthesis of high quality multi-crystalline films, some complex problems have only been partially mitigated^3^. The often observed hysteretic behavior during *J–V* characterization, caused by ion mobility, can be considered a primary indication for the intrinsic long-term steady-state instability^9^. In that respect, the phase stabilization of specific perovskite formulations has been the object of thorough investigation^2,10,11^. Recently, the reduction of defects at the surface and grain boundaries of the perovskite film as well as at the interfaces with the electrical contacts, has attracted great interest for its dramatic impact on the operational stability and efficiency of the device^7,12^. Mitigation of surface defects, whether at the interface of perovskite/HTL or perovskite/electron transport layer (ETL), provides the added benefit of improving the open-circuit voltage (*V*_oc_) without affecting the charge carrier transport or the fill factor (FF). Previous studies have employed several types of ammonium cations in order to impede the charge carrier recombination losses occurring at the interfaces or throughout the bulk of the perovskite film. Among those, formamidinium bromide (FABr) was used as an electron blocking layer, forming a wide band gap over layer (FAPbBr_3-x_I_x_) at the interface between the perovskite/HTL and consequently improving *V*_oc_ by approximately 60 mV^13^. In a similar manner methylammonium iodide (MAI) was thermally evaporated at the interface perovskite/HTL, enhancing efficiency from 14.5% to 17.2% with a high reproducibility^14^. Lately, quaternary ammonium halides were found to decrease the ionic defects at the perovskite surface and significantly improve efficiency and stability^7^. Furthermore, phenylalkylamine molecules^15,16^, and polymers^17^ have been used to improve the efficiency and moisture tolerance of perovskite solar cells (PSC). Moreover, guanidinium^18–21^, ethylammonium^22,23^, and imidazolium^24,25^ have been used as additive to improve different aspects of PSC operation.

**Fig. 1:**
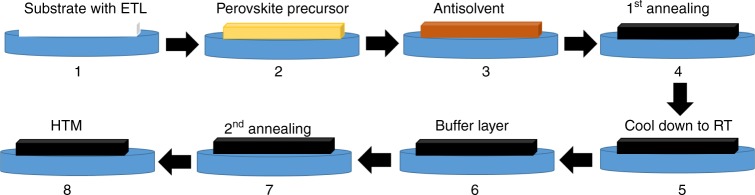
Scheme of our method for treating perovskite films by spin-coating of different organic ammonium salts (EAI, IAI, and GuaI) and consecutive annealing

Herein we report a facile strategy to tailor the interface between the perovskite and the HTL (Fig. 1). We show that the modification of the perovskite surface via the addition of organic ammonium salts, namely, ethylammonium iodide ([(C_2_H_5_)NH_3_]I, (EAI)), imidazolium iodide ([C_3_N_2_H_5_]I, (IAI)), and guanidinium iodide ([C(NH_2_)_3_]I, (GuaI)), considerably increases the device performance. We use mixed-cation/halide perovskite formulations of the composition (FA_0.9_Cs_0_._07_MA_0_._03_Pb(I_0_._92_Br_0_._08_)_3_) with 3% excess of PbI_2_. These agents greatly reduce the hysteresis in the *JV* curve improving the solar to electric power conversion efficiency from 20.5% for the control device to 22.3, 22.1, and 21.0% for the EAI-, IAI-, and GuaI-treated device, respectively. Moreover, defect mitigation improves the operational stability for the PSC’s, which was tested at full solar intensity under maximum power tracking condition for 550 h with a small loss of only 5% for the best performing devices.

## Results

### Characterization and fabrication of perovskite thin films

We employed a device architecture comprising an FTO glass substrate, on top of which we deposited a compact TiO_2_ layer followed by mesoporous TiO_2_/perovskite/passivation layer/spiro-OMeTAD/gold. The complete procedure for the device fabrication is detailed in the method section. In short, the perovskite film was annealed at 150 °C for 30–40 min. After cooling down to room temperature, the passivation layer was immediately deposited by spin-coating a solution of ammonium salt (EAI, IAI, and GuaI) in isopropanol and subsequent annealing at 70 °C for 10–15 min. The concentration of the solution was optimized for each compound investigated (Supplementary Table 1) and the different treatments were compared at their best condition. We note that the high annealing temperature could potentially drive off methylammonium from the thin film. Solid-state NMR quantification^26^ of the cation content of our films yielded 0.97 mol % FA and 0.03 mol % MA, which agrees with the stoichiometry of the precursor solution, indicating that MA is fully retained in the final perovskite composition even after the high annealing (Supplementary Fig. 1 and Supplementary Note 1).

**Fig. 2:**
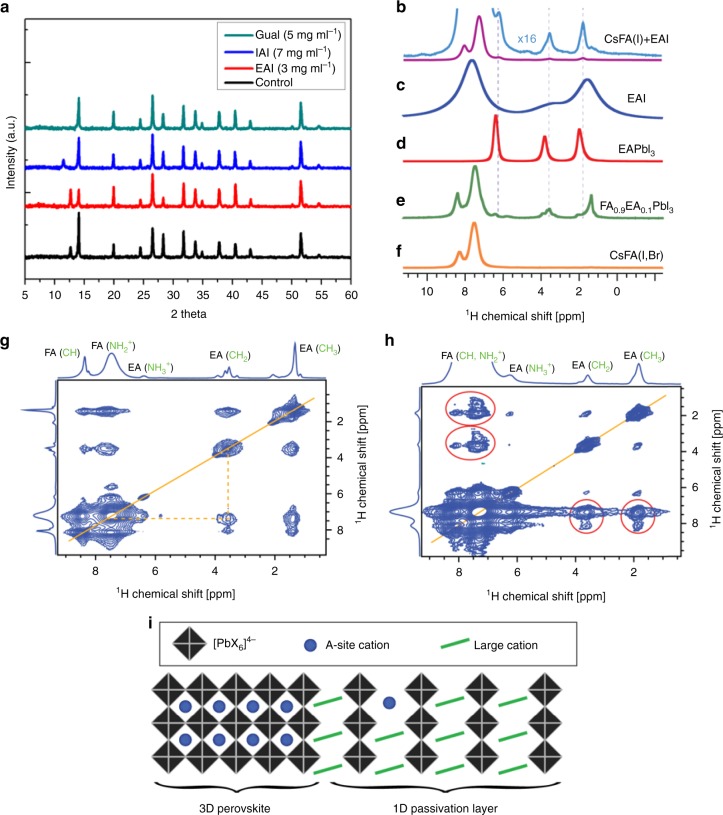
Structural characterization of the passivated perovskite films: **a** XRD patterns of the treated films compared to the control. Solid-state ^1^H MAS NMR measurements at 21.1 T, 300 K and 20 kHz MAS (unless noted otherwise): **b** thin film of CsFA(I) treated with 5 mg ml^−1^ EAI (at 60 kHz MAS), **c** neat EAI, **d** bulk mechanochemical EAPbI_3_, (**e**) bulk mechanochemical FA_0.9_EA_0.1_PbI_3_, **f** thin film of FA_0.93_Cs_0.07_Pb(I_0.92_Br_0.08_)_3_ (CsFA(I,Br)), **g** ^1^H–^1^H spin diffusion (20 kHz MAS) experiment evidencing atomic-level proximity between FA and EA in the mixed FA/EA phase; one of the EA/FA cross-peaks has been indicated with dashed lines. **h** ^1^H–^1^H spin diffusion experiment (60 kHz MAS) evidencing atomic-level proximity between FA and EA in the FA_0.93_Cs_0.07_PbI_3_, thin film treated with 5 mg ml^−1^ EAI (FA/EA cross-peaks in red circles), **i** schematic representation of the 1D/3D heterostructure evidenced by solid-state NMR proximity measurements

The surface treatment described above significantly modifies the perovskite composition and surface morphology due to a chemical reaction with the perovskite. This was traced by crystallographic structure analysis of the perovskite film using X-ray diffraction (XRD). Fig. 2a and Supplementary Fig. 2a, b, c, reveal the presence of unreacted PbI_2_ (2*θ* = 12.7°) which is expected, since the precursor solution contains 3% excess of PbI_2_. All three surface treatments have a similar effect on the XRD pattern. In particular, for concentrations above 3 mg/ml for all treatments, the PbI_2_ peaks completely disappear (Fig. 2a and Supplementary Fig. 2a–c), presumably via formation of a thin non-perovskite cover layer of EA/IA/Gua lead halide on top of the perovskite film. We have recently shown that solid-state magic angle spinning (MAS) NMR can be used to probe this kind of atomic-level microstructure of multi-component lead halide perovskites^21,26–28^. We therefore test the hypothesis by carrying out solid-state NMR measurements on a thin film of FA_0.93_Cs_0_._07_PbI_3_ (further referred to as CsFA(I)) treated with EAI (5 mg ml^−1^). We used the pure-iodide CsFA(I) composition to avoid the overlap between MA and EA ^1^H signals and the formation of mixed iodide-bromide phases, which would complicate the interpretation. Figure 2b shows a ^1^H solid-state MAS NMR spectrum of the CsFA(I) thin film treated with 5 mg ml^−1^ EAI and identifies the presence of two organic cations in the film: FA (8.1 ppm (CH) and 7.3 ppm (NH_2_^+^)) as well as EA (6.3 ppm (NH_3_^+^), 3.6 ppm (CH_2_) and 1.8 (CH_3_)). There is no unreacted EAI in the film (Fig. 2c), which would give signals at 7.7, 3.3, and 1.6 ppm with a full width at half maximum (FWHM) of 1.5–2.0 ppm due to strong ^1^H–^1^H dipole-dipole couplings. The EA signals in the thin film have FWHM of 0.1–0.2 ppm, consistent with lower ^1^H density in the EA-containing phase^29^. The new EA species in the thin film have a spectral signature similar to that of EAPbI_3_ (Fig. 2d, 6.4, 3.8, and 2.0 ppm). We thus confirm that EAI is fully converted into EA-containing lead halide phases during the passivation treatment.

That said, the EA signals are slightly shifted, indicating a small structural difference with respect to the pure 1D EAPbI_3_ phase^29^.This can be potentially caused by the formation of mixed FA/EA, Cs/EA, and/or Cs/EA/FA phases. We demonstrate this by preparing α-FAPbI_3_ substitutionally doped with 10 mol% EAI (FA_0.9_EA_0.1_PbI_3_) (Fig. 2e). The spectrum of this material shows a distribution of environments for each of the EA sites (CH_3_: 0.6–2.3 ppm, CH_2_: 3.0–4.3 and NH_3_^+^: 5.3–6.7 ppm). This distribution is likely caused by the formation of mixed FA/EA structures with a varying number of FA slabs separated by EA spacers, in structures similar to those reported for 2-(*1H*-pyrazol-1-yl)pyridine-doped 1D/3D^30^ and butylammonium- and phenylethylammonium-based 2D/3D materials^31^. We further evidence the formation of mixed FA/EA phases by performing two-dimensional ^1^H–^1^H spin diffusion experiments, which correlate signals based on their spatial proximity (up to around 10Å)^32^ (Fig. 2g). The peaks lying on the diagonal correspond to the species presented in the 1D projection above. On the other hand, off-diagonal peaks indicate that two chemical environments are in atomic-level proximity (an example is illustrated by the dashed orange line). Beside trivial intramolecular contacts, every EA environment (CH_3_, CH_2_ and NH_2_) is correlated to each of the two FA environments (CH and NH_2_^+^), demonstrating unambiguously that EA and FA are microscopically mixed within the same phase.

We then carried out the ^1^H–^1^H spin diffusion measurement on the EAI-treated CsFA(I) thin film and found through-space atomic-level contact between FA and EA (Fig. 2h, red circles), confirming that the formation of mixed EA/FA phases is general, regardless of the processing conditions. We also show that analogous atomic-level proximities are present in the case of IAI and GuaI treatment, confirming the formation of 3D/1D heterostructures in these cases (Supplementary Figs. 7h–m and 8). While the exact spectral signature of EA in such mixed phases will depend on the FA/EA ratio and can conceivably be further modified by the presence of Cs, the chemical shift range and line widths corresponding to the model FA/EA phase matches that observed in the EAI-passivated thin film of CsFA(I). This finding is of paramount importance in that it shows that EAI is fully converted into new mixed EA/FA phases. Since the reaction is aided by IPA and as such happens without redissolution and recrystallization of the perovskite, the new EA/FA phase must form on the surface of the preexisting perovskite grains and its similarity from NMR to the 1D EAPbI_3_ suggests it is a 1D structure. We note that it is not possible to quantify the EA/FA ratio in the 1D passivation layer by simply comparing it to reference 1D FA_1-x_EA_x_PbI_3_ phases (Supplementary Fig. 7c–g), as in the 3D/1D heterostructure the EA shift is additionally affected by the presence of the 3D perovskite phase in its immediate microscopic environment. This supports the formation of a firmly adhering passivation layer with high ambient stability, owing to the superior stability of 3D perovskites in hybrids with lower dimensionality structures (Fig. 2i)^31^ We also provide a comparison with a EA-treated film but without the second annealing step, which shows that the final 3D/1D heterostructure is formed during spin coating (Supplementary Fig. 7a–b). Finally, we note that solid-state ^1^H MAS NMR quantification of the cation content in the passivated thin film revealed that the EA constitutes 10 mol % of the total organic cation content (with 90 mol % FA). After applying straightforward chemical and geometrical consideration, this corresponds to 27 nm thick 1D passivation layer (Supplementary Note 2).

**Fig. 3:**
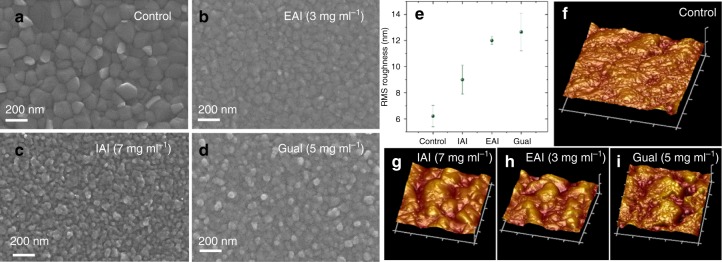
Morphological characterization of perovskite layers: **a**–**d** SEM top view images of the perovskite films with optimized treatment conditions. **e** AFM measurements (**x**–**y**: 1 × 1 μm, z: 0 ± 90 nm) reveal increasing RMS surface roughness for surface-treated perovskite absorbers: **f** control, **g** IAI, **h** EAI, and **i** GuaI

Furthermore, surface morphology of the perovskite films was recorded via scanning electron microscopy (SEM) (Fig. 3a–d). In this study, the bulk perovskite composition was kept identical for all the conditions and only the surface was modified by the treatment with the 3 different agents. From Fig. 3a–d and Supplementary Fig. 3 we infer that the surface of the treated films shows much smaller grains than the control sample. To rule out etching of the perovskite surface by IPA as a cause of the roughening, we examined a control film, which was treated by neat IPA and ascertained that the solvent on its own does not modify the perovskite surface morphology (Supplementary Fig. 3). We then investigate surface roughness of the perovskite films after the surface treatment using atomic force microscopy (AFM) (Fig. 3f–j). It is apparent that the surface roughness, and thereby the corresponding specific surface area, has increased significantly for all the treated samples, with GuaI (5 mg ml^−1^) showing the highest root mean square (RMS) roughness of 12.7 nm, followed by EAI (3 mg ml^−1^) with 12.0 nm, IAI (7 mg ml^−1^) with 9.00 nm, and the control with 6.21 nm.

**Fig. 4:**
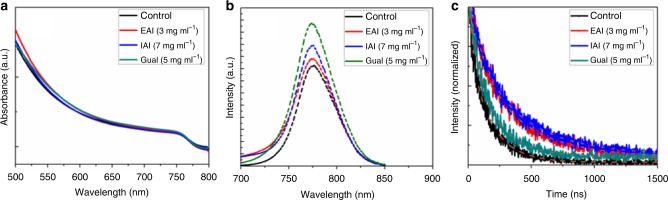
Optical properties of perovskite films: **a** Absorbance, **b** steady-state photoluminescence, and **c** time-resolved photoluminescence spectra of treated and untreated perovskite films; dashed lines present the fitting curves

Figure 4a and Supplementary Fig. 4 show that the absorbance onset for control/IPA corresponded to that of EAI, IAI, and GuaI-passivated films indicating that the band gap of the bulk perovskite was not noticeably affected by the surface treatment. We investigated the steady-state and time-resolved photoluminescence (PL) of the control and modified perovskite layer. Fig. 4b and Supplementary Fig. 1a–d show an increase in PL intensity in response to the post treatments. This suggests a reduction of the non-radiative recombination losses that could be explained by defect mitigation induced by cation exchange and filling of iodide vacancies at the absorber surface in agreement with previous work on methylammonium lead iodide films^14^. It is worth mentioning that the GuaI when applied at 7 mg ml^−1^ and 10 mg ml^−1^ showed a slight red shift as shown in Supplementary Fig. 5.

We carried out time-resolved photoluminescence (TRPL) on glass/Al_2_O_3_/perovskite samples with and without surface treatment. We excited the sample from the from the perovskite side using 670 nm wavelength light. By fitting the luminescence decays in Fig. 4c according to our previously reported procedure^27^, we derived lifetimes *τ*_1_ = 1/*k*_1_ for the pseuo-first order trap mediated (Shockley–Read-Hall) non-radiative PL decay process. From TRPL data in Fig. 4c, the decay lifetime increased with the application of our surface treatment from 250 ns for the control to 560 ns, 625 ns, and 333 ns for EAI (3 mg ml^−1^), IAI (7 mg ml^−1^), and GuaI (5 mg ml^−1^), respectively.

### Photovoltaic device and performance

**Fig. 5:**
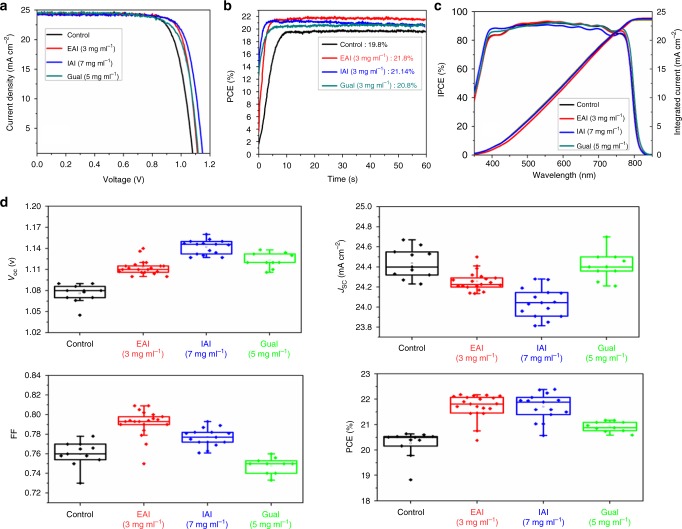
Photovoltaic characterization: **a** *J-V* curves of the devices with different passivation layers in comparison to the control device without any treatment, with a scan rate of 10 mV/s **b** Maximum power tracking of the same device, **c**) IPCE spectra and calculated photocurrent integrated over the standard AM 1.5 G solar emission. In agreement to the *J-V* data no change in the spectra is observed, and **d** *J–V* metrics of perovskite devices with different treatment in comparison to the control

We examined the effect of surface passivation by ammonium salts on the photovoltaic performance for complete devices in a FTO/c-TiO_2_/m-TiO_2_/perovskite/spiro-OMeTAD/Au configuration (for more details see Methods). The data are shown in Fig. 5a in comparison to a control device without passivation (see Supplementary Table 1 for a comparison of all treatments with different concentrations).

The open-circuit voltages of all treated devices are significantly increased with an average improvement of 30 mV, 70 mV, and 40 mV, for EAI, IAI, and GuaI, respectively (see Fig. 5d). While the GuaI-treated devices showed an average reduction of the FF by 2% absolute, the FF increased by 3.5 and 2% for EAI and IAI-treated devices, respectively, reaching up to 81% on the best performing EAI device without significant loss of the short circuit current (*J*_sc_). The short circuit photocurrent densities are barely modified with a maximum average decrease of 0.4 mA cm^−2^ for the IAI-treated devices. Overall, the mean efficiency increased from 20.5% for the control device to the 22.3%, 22.1%, and 21.0% for EAI, IAI, and GuaI-treated devices, respectively, with the champion device treated via EAI achieving up to 22.3% PCE. Figure 5b further confirms stable power output for all treated devices during maximum power point tracking under one sun illumination for one minute. The incident photon-to-current efficiency (IPCE) and integrated current density as a function of wavelength are shown in Fig. 5c. In agreement to UV-Vis, no shift in the onset of the IPCE spectra is detected for treated devices while integrated current densities agree well with the *J*_*s*c_ -values derived from the *JV* measurements. Moreover, Supplementary Fig. 6a–d and Supplementary Table 2 show that the use of these ammonium salts as surface treatment is capable of reducing the hysteresis. To further analyze the effect of these buffer layers on the electrical properties, we performed intensity modulated voltage spectroscopy (IMVS). The data shown in Supplementary Fig. 6e agree with the trend found for *V*_oc_, i.e., the devices showing higher *V*_oc_ show a longer electron lifetime at *V*_oc_.

### Operational stability of perovskite solar cells

We also investigated is the operational stability of our PSCs under working conditions. This remains a major concern for PSC, which needs to be urgently addressed^33^. Best performing devices were subjected to full sunlight intensity with maximum power tracking for 550 h at room temperature in a nitrogen atmosphere.

**Fig. 6:**
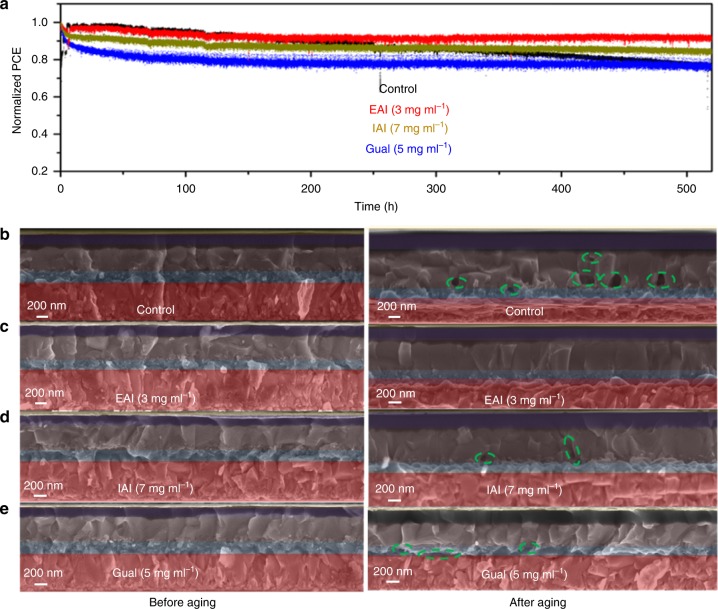
Stability data and cross section before and after aging for control and treated devices: **a** A comparison of operational stability of control and treated perovskite devices. The devices were measured under a nitrogen environment at room temperature under constant illumination (LED source, ~1 Sun) at a maximum power point for 550 h. **b**, **c**, **d**, **e** Cross-sectional SEM images of the control and modified perovskite devices before and after aging at room temperature under constant illumination (LED source, ~1 Sun) for 550 h. Images show perovskite absorber and covering HTL

We note the good stability of the control device preserving 80% of its initial PCE value. This is attributed to the perovskite composition employed in this work that has an extremely low content of MAI and contains a small addition of cesium iodide (CsI) to stabilize the predominately formamidinium lead iodide perovskite phase. From Fig. 6a, the passivated devices show a different behavior, a small initial performance drop being followed by a stable power output. Remarkably for the EAI-treated device the overall loss in PCE is only 5%. By contrast the control device shows a small but constant drop over the whole duration of the test. It is well-established that this behavior is due to the migration of ions from the HTM (LiTFSI) and gold from the gold electrode into the perovskite layer and to the TiO_2_ working electrode^34^. The fact that the efficiency loss for the passivated devices ceases after an initial decrease, it is reasonable to assume that that the passivating layer introduces a barrier at the HTM/perovskite interface that prevents the diffusion of HTM additives and gold diffusion into the perovskite layer. From cross-sectional SEM images (Fig. 6b–e) we can see that before aging both control and modified devices appeared to have compact absorber layers. After 550 h or MPP testing, however, the appearance of voids at the interface between the mesoporous TiO_2_ and perovskite in the control and few voids also in the IAI- and GuaI-treated perovskites is visible. These voids, stemming from the degradation of the perovskite absorber, may affect the photon absorption and act as recombination centers to hinder charge carrier collection during operation of the solar cell. Confirming this trend, EAI showed good stability at MPP under constant illumination with a decrease of only 5% and the absence of voids in the cross section SEM image of the aged devices shown in Fig. 6c.

## Discussion

In summary, we investigated the effect of different ammonium salts (i.e., EAI, IAI, and GuaI) as surface passivation agents on mixed-cation and mixed-halide perovskite films. Solid-state NMR has evidenced that they form a tightly adhering low-dimensionality passivation layer of on the preexisting perovskite grains. The introduction of these buffer layers showed a significant enhancement of the open-circuit voltage by 30 mV (EAI), 70 mV (IAI), and 40 mV (GuaI), leading to the realization of PCEs as high as 22.3%, 22.1%, and 21.0% for EAI, IAI, and GuaI-treated devices, respectively. EAI and IAI showed an improvement also on the FF (+3.5% and +2%, respectively) with EAI reaching a FF of 81% on the best performing device. In contrast to the control sample, all passivated devices, after an initial drop, stabilized their efficiency with the EAI-treated device only losing 5% of its initial value after 550 h of MPP tracking. This work exemplifies the importance of interface engineering for perovskite solar cells and should stimulate other successful developments in the future.

## Methods

### Materials

All materials were purchased from Sigma-Aldrich and used as received, unless stated otherwise.

### Solar cell preparation

Fluorine-doped tin oxide (FTO)-glass substrates (TCO glass, NSG 10, Nippon sheet glass, Japan) were cleaned by ultrasonication in Hellmanex (2%, deionized water), rinsed thoroughly with deionized water and ethanol, and then treated in oxygen plasma for 15 min. Thirty nanometer blocking layer (TiO_2_) was sprayed on the cleaned FTO by at 450 °C using a commercial titanium diisopropoxide bis(acetylacetonate) solution (75% in 2-propanol, Sigma-Aldrich) diluted in anhydrous ethanol (1:9 volume ratio). A 150 nm mesoporous TiO_2_ layer (diluted paste (1:6 wt. ratio) (Dyesol 30NRD: ethanol)) spin coated at 5000 rpm for 15 s, and then sintered at 450 °C for 30 min in dry air.

### Synthesis of perovskite films

The perovskite films were deposited using a single-step deposition method from the precursor solution, which was prepared in Argon atmosphere and containing 1.35 M of FAI, FABr, MAI, CsI, PbI_2_ and PbBr_2_ in anhydrous dimethylformamide/ dimethylsulphoxide (4:1 (volume ratio)) to achieve the desired composition: FA_0.9_Cs_0.07_MA_0.03_Pb(I_0.92_Br_0.08_)_3_ (3% PbI_2_ excess). The device fabrication, including the surface treatment step, was carried out inside a dry air box, under controlled atmospheric conditions with humidity <2%.Perovskite solution was spin-coated in a two-step program at 1000 and 6000 rpm, respectively. Two hundred microliter of chlorobenzene was dropped on the spinning substrate. This was followed by annealing the films at 150 °C for 30–40 min. After preparing the initial perovskite layer (control) as described above, the film was cooled down at room temperature. Then, the surface treatment was performed by spin-coating a EAI, IAI, and GuaI-solution in isopropanol at different concentrations of 0 mg ml^−1^, 3 mg ml^−1^, 5 mg ml^−1^, 7 mg ml^−1^, and 10 mg ml^−1^ at 6000 rpm for 30 s, followed by annealing at 70 °C for 10~15 min. For completing the fabrication of devices, 85 mg of 2,2′,7,7′-tetrakis(*N*,*N*-di-*p*-methoxyphenylamine)-9,9-spirobifluorene (spiro-OMeTAD) was dissolved in 1 ml of chlorobenzene as a hole-transporting material (HTM). The HTM was spin coated at 4000 rpm for 20 s. The HTM was doped with bis(trifluoromethylsulfonyl)imide lithium salt (17.8 µl prepared by dissolving 520 mg LiTFSI in 1 ml of acetonitrile), and 28.8 µl of 4-tert-butylpyridine. Finally, a ~80 nm gold (Au) layer was thermally evaporated.

### Device characterization

The current-voltage (*J–V*) characteristics of the perovskite devices were recorded under ambient temperature and air conditions with a digital source meter (Keithley model 2400, USA). A 450 W xenon lamp (Oriel, USA) was used as the light source for photovoltaic (*J–V*) measurements. The spectral output of the lamp was filtered using a Schott K113 Tempax sunlight filter (Präzisions Glas & Optik GmbH, Germany) to reduce the mismatch between the simulated and actual solar spectrum to less than 2%. The photo-active area of 0.16 cm^2^ was defined using a dark-colored metal mask.

### Incident photon-to-current efficiency (IPCE)

It was recorded under a constant white light bias of approximately 5 mW cm^−2^ supplied by an array of white light emitting diodes. The excitation beam coming from a 300 W Xenon lamp (ILC Technology) was focused through a Gemini- 180 double monochromator (Jobin Yvon Ltd) and chopped at ~2 Hz. The signal was recorded using a Model SR830 DSP Lock-In Amplifier (Stanford Research Systems).

### Scanning electron microscopy (SEM)

It was performed on a ZEISS Merlin HR-SEM.

### Atomic force microscopy (AFM)

AFM images were obtained using a Bruker Dimension Icon Atomic Force Microscope in tapping mode.

X-ray powder diffractions were recorded on an X’Pert MPD PRO (Panalytical) equipped with a ceramic tube (Cu anode, λ = 1.54060 Å), a secondary graphite (002) monochromator and a RTMS X’Celerator (Panalytical).

UV–Vis measurements (Uv-Vis) were performed on a Varian Cary 5.

Photoluminescence spectra (PL) were obtained with a Florolog 322 (Horiba Jobin Ybon Ltd) in the wavelength range from 500 to 850 nm by exciting at 460 nm.

Time-resolved photoluminescence (TRPL) was measured with a spectrometer (FluoroLog-3, Horiba) working in a time-correlated single-photon counting mode with less than ns time resolution. A picosecond pulsed diode laser head NanoLED N-670L (Horiba) emitting less than 200 ps duration pulses at 670 nm with a maximum repetition rate of 1 MHz was used as excitation source. The measurements were carried out under ambient conditions and no change in PL was observed during the course of the measurements (several hours) indicating no appreciable decomposition.

### Solid-state NMR measurements

Room temperature ^1^H (900.0 MHz) NMR spectra were recorded on a Bruker Avance Neo 21.1 T spectrometer equipped with a 3.2 mm and 1.3 mm CPMAS probe. ^1^H chemical shifts were referenced to solid adamantane (*δ* = 1.91 ppm). Quantitative ^1^H spectra were acquire with a recycle delay of 150 s. The CsFA and CsMAFA samples were prepared as thin films on glass slides using the same deposition technique as for PSC fabrication and scratched off the slides into a rotor. EAPbI_3_ and FA_0.9_EA_0.10_PbI_3_ were prepared using mechanosynthesis, according to previously published procedures^21,26,35–37^. ^1^H–^1^H spin diffusion measurements at 20 kHz MAS were carried out using a mixing period of 50 ms and a recycle delay of 1 s (FA_0.90_EA_0.10_PbI_3_). ^1^H–^1^H spin diffusion measurements at 60 kHz MAS used a mixing period of 4 s and a recycle delay of 3 s (CsFA(I) thin film).

IMVS measurements were performed by Bio-Logic SP300 in combination with the Galvano Staircase Spectroscopy routine from EC-Lab Software. Further description of the technique is provided in Supplementary Note 2.

### Long-term light soaking test

Stability measurements were performed with a Biologic MPG2 potentiostat under a full AM 1.5 Sun-equivalent white LED lamp. The devices were measured with a maximum power point (MPP) tracking routine under continuous illumination at room temperature. The MPP was updated every 10 s by a standard perturb and observe method. Every minute a *JV* curve was recorded in order to track the evolution of individual *JV* parameters.

## Supplementary information

Supplementary Information

Source Data

## Data Availability

Data that support the findings of this study are available in separate Supplementary Data Files in Supplementary Information section. All other relevant data are available from the corresponding authors upon reasonable request.
