# Supplementary material for: Atomic-level passivation mechanism of ammonium salts enabling highly efficient perovskite solar cells
Source: Nat Commun. 2019 Jul 8;10:3008. doi: 10.1038/s41467-019-10985-5 (PMC6614363; doi:10.1038/s41467-019-10985-5)
Supplement: Supplementary file 1 — Supplementary Information [file 41467_2019_10985_MOESM1_ESM.pdf]

Supplementary information

Atomic-level passivation mechanism of ammonium salts enabling highly efficient perovskite solar cells, Alharbi et al.

Supplementary information

Atomic-level passivation mechanism of ammonium salts enabling highly efficient perovskite solar cells

Essa A. Alharbi [1], Ahmed Y. Alyamani [2], Dominik J. Kubicki [1,3], Alexander R. Uhl [1], Brennan J. Walder [3], Anwar Q. Alanazi [1] , Jiangshan Luo [1,4] , Andrés Burgos-Caminal [4], Abdulrahman Albadri [2], Hamad Albrithen [2,5], Mohammad Hayal Alotaibi [2], Jacques-E. Moser[45], Shaik M. Zakeeruddin [1], Fabrizio Giordano [1], Lyndon Emsley [3]* and Michael Grätzel [1]**

[1] Laboratory of Photonics and Interfaces, Institute of Chemical Sciences and Engineering, School of Basic Sciences, Ecole Polytechnique Fédérale de Lausanne, CH-1015 Lausanne, Switzerland.

[2] National Center for Nanotechnology, King Abdulaziz City for Science and Technology, P.O. Box 6086, Riyadh 11442, Saudi Arabia.

[3] Laboratory of Magnetic Resonance, Institute of Chemical Sciences and Engineering, School of Basic Sciences, Ecole Polytechnique Fédérale de Lausanne, CH-1015 Lausanne, Switzerland.

[4] Photochemical Dynamics Group, Institute of Chemical Sciences and Engineering, Lausanne Centre for Ultrafast Science, École polytechnique fédérale de Lausanne CH-1015 Lausanne Switzerland.

[5] Physics and Astronomy Department-Research Chair for Tribology, Surface and Interface Sciences, College of Science, and King Abdullah Institute for Nanotechnology-Aramco Laboratory for Applied Sensing Research, King Saud University, P.O. Box 2455, Riyadh 11451, Saudi Arabia.

Present affiliation:

[#] Laboratory for Solar Energy and Fuels, The University of British Columbia, Kelowna, BC, V1V 1V7, Canada.

[†] Institute of Photoelectronic Thin Film Devices and Technology, Key Laboratory of Photoelectronic Thin Film Devices and Technology of Tianjin, Nankai University, Tianjin, 300350 China.

** Correspondence should be addressed to fabrizio.giordano@epfl.ch, lyndon.emsley@epfl.ch or michael.graetzel@epfl.ch.*

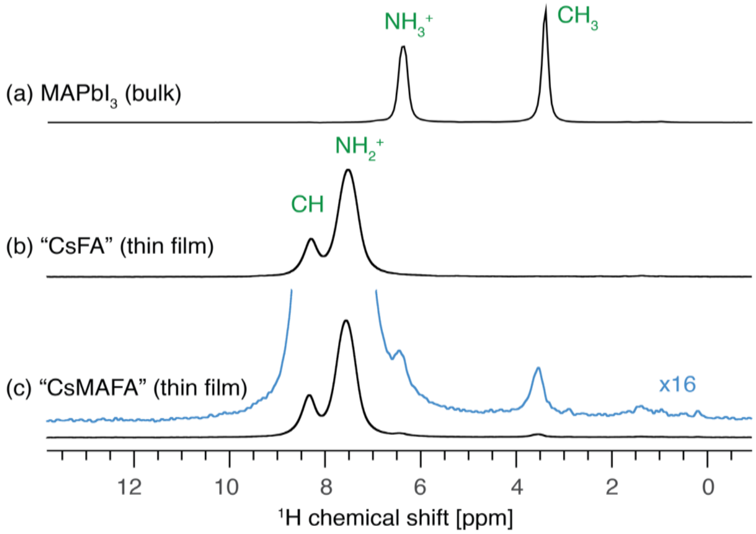

Supplementary Figure S1. ^{1}H solid-state MAS NMR spectra at 21.1 T, 298 K and 20 kHz MAS of (a) bulk mechanochemical $MAPbI_3$, (b) the $FA_{0.93}Cs_{0.07}Pb(I_{0.92}Br_{0.08})_3$ ("CsFA(I,Br)") composition prepared as a thin film, (c) the $FA_{0.9}Cs_{0.07}MA_{0.03}Pb(I_{0.92}Br_{0.08})_3$ ("CsMAFA") composition prepared as a thin film (bottom: normalised intensity, top: intensity multiplied by 16 toevidence the MA signals).

.

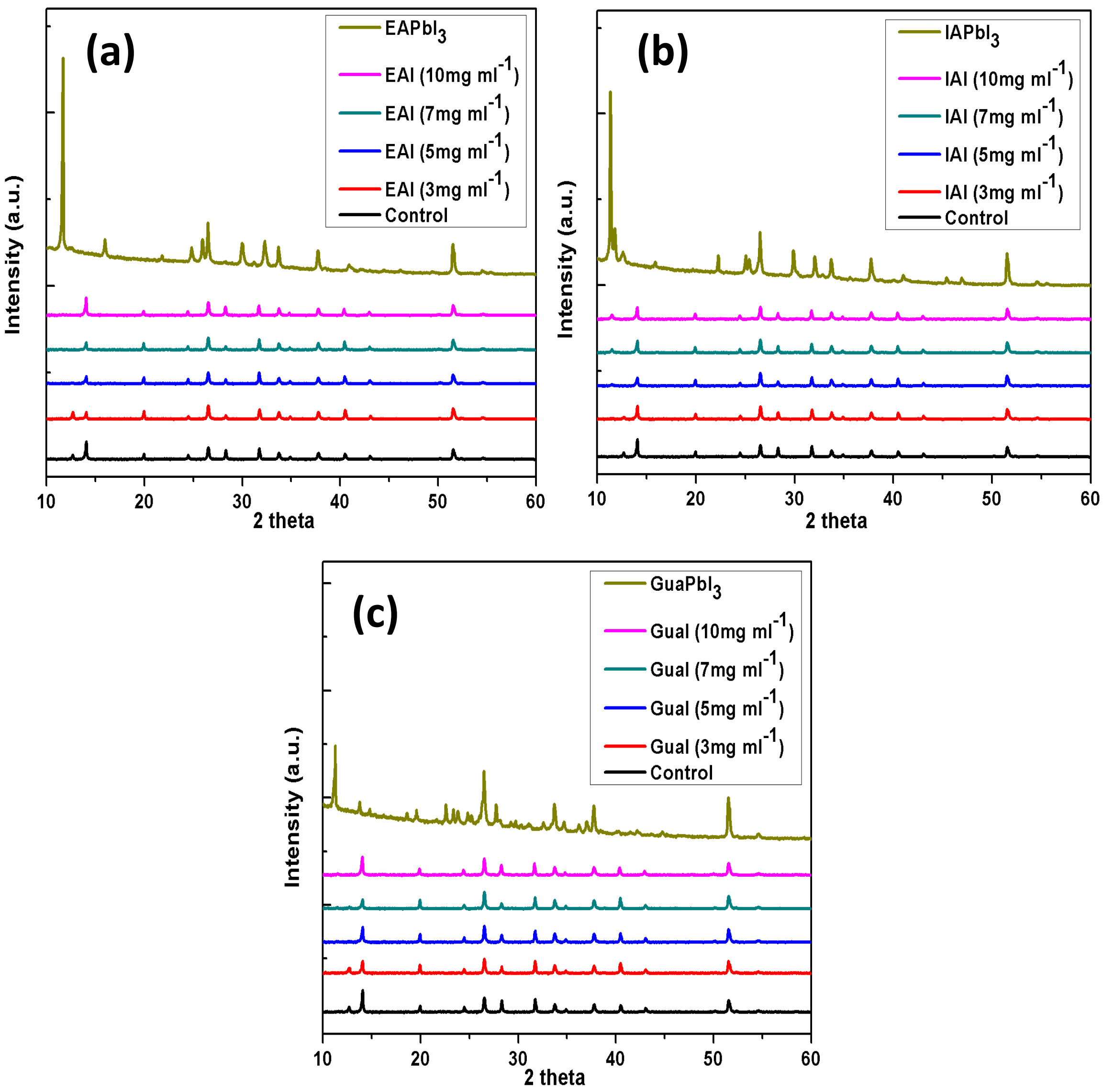

Supplementary Figure S2. XRD patterns of a) EAI, b) IAI and c) GuaI (Control film and $EAPbI_3$, $IAPb_3$ and $GuaPbI_3$ including with each accordingly)

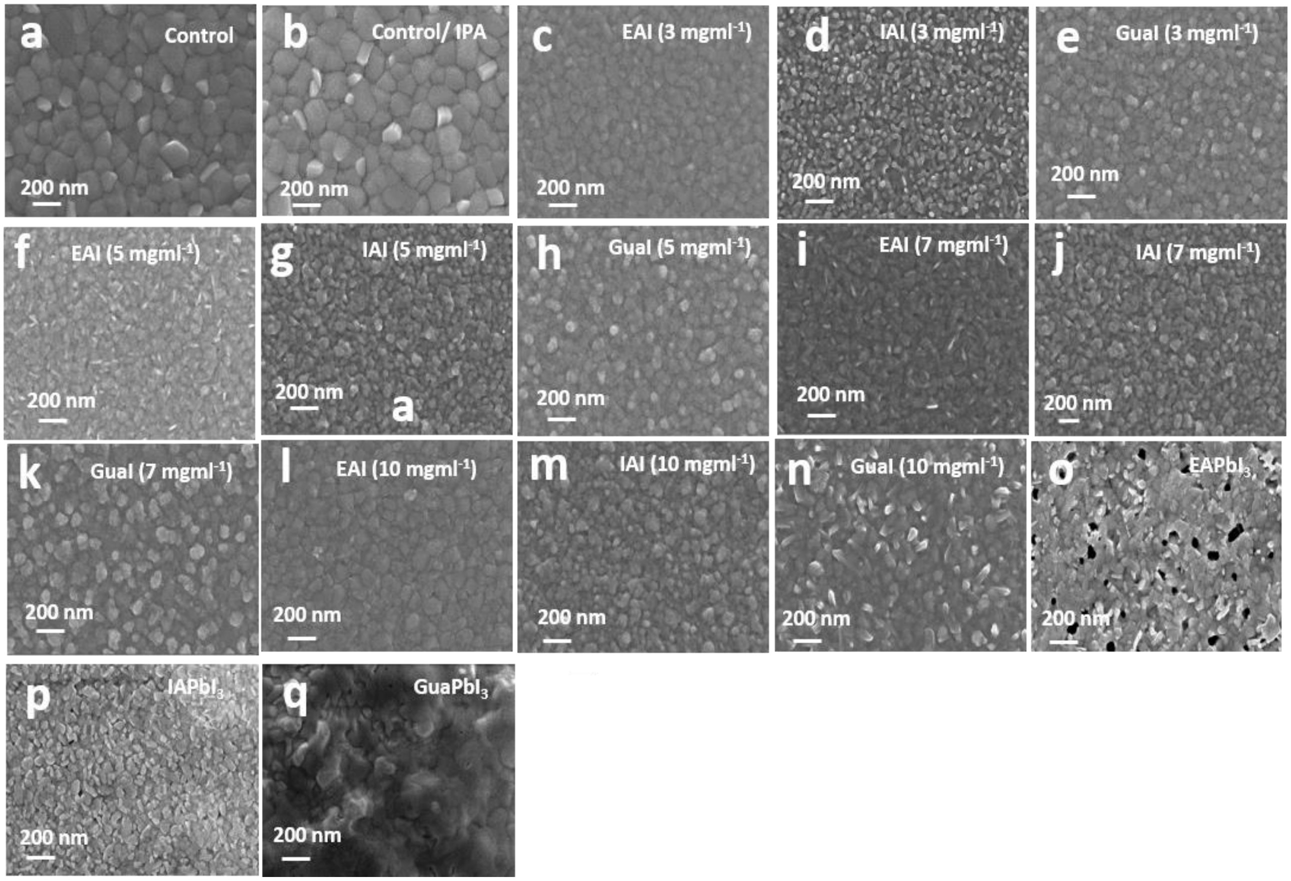

Supplementary Figure S3. Top view SEM micrographs of Control, EAI, IAI and GuaI respectively, at different concentration (from 0-10 mg/ml) and $EAPbI_3$, $IAPb_3$ and $GuaPbI_3$.

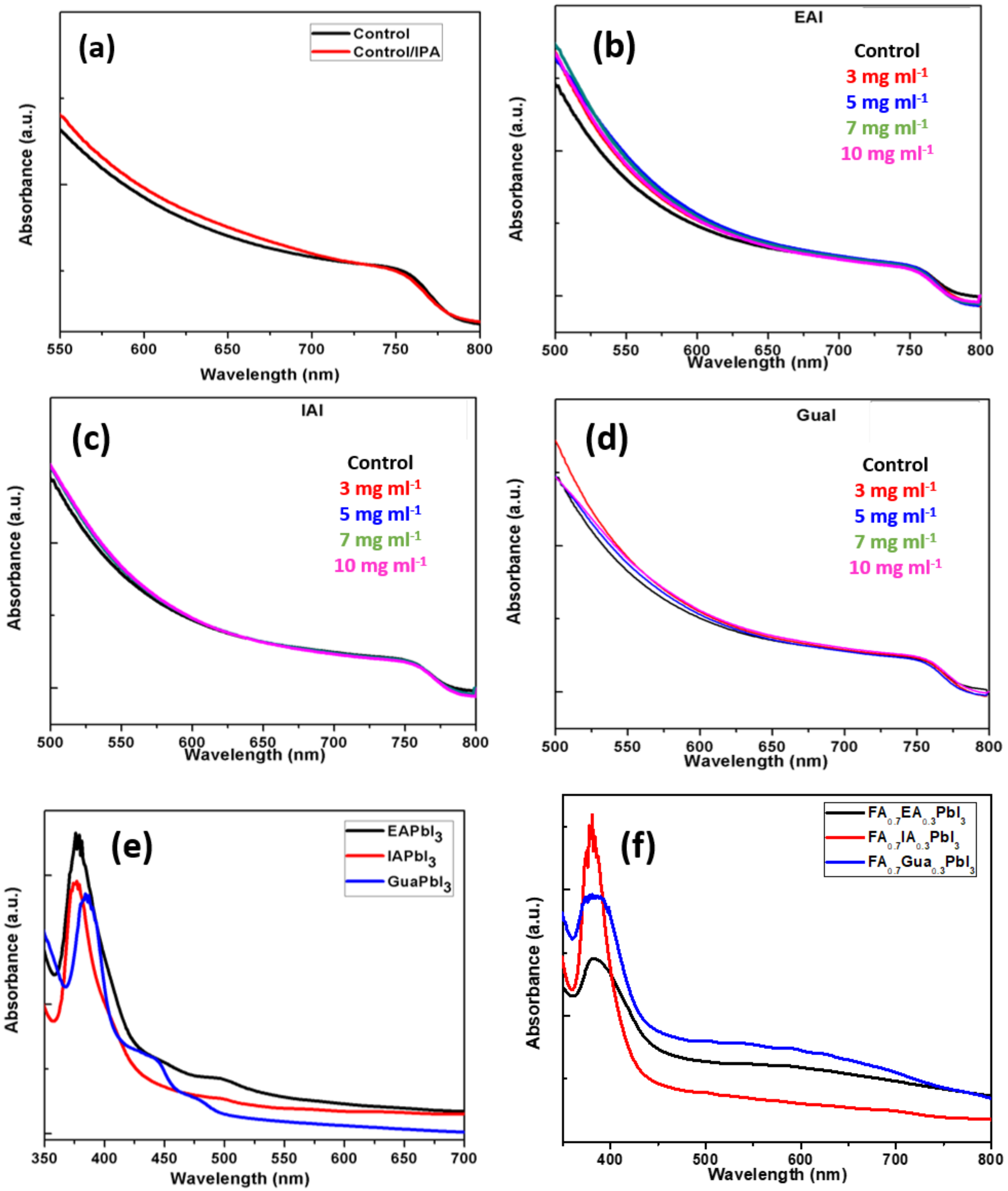

Supplementary Figure S4. A comparison of UV-Vis of a)Control/IPA b) EAI, c) IAI, d) GuaI, e) $EAPbI_3$, $IAPbI_3$ and $GuaPbI_3$ and (f) $FA_{0.70}X_{0.30}PbI_3$ (X=EAI, IAI and GuaI).

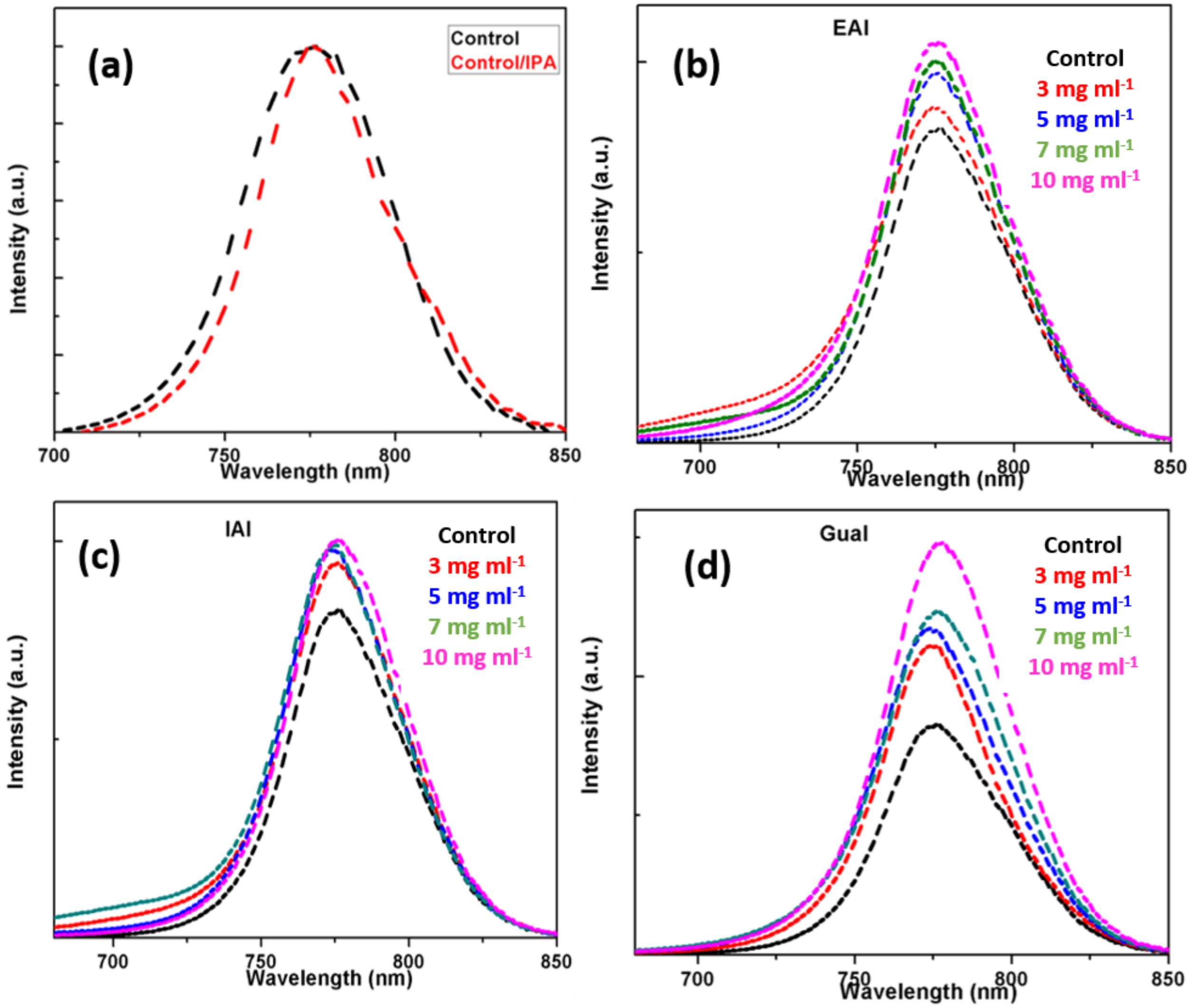

Supplementary Figure S5. Photoluminescence studies of a) Control/IPA b) EAI, c) IAI and d) GuaI.

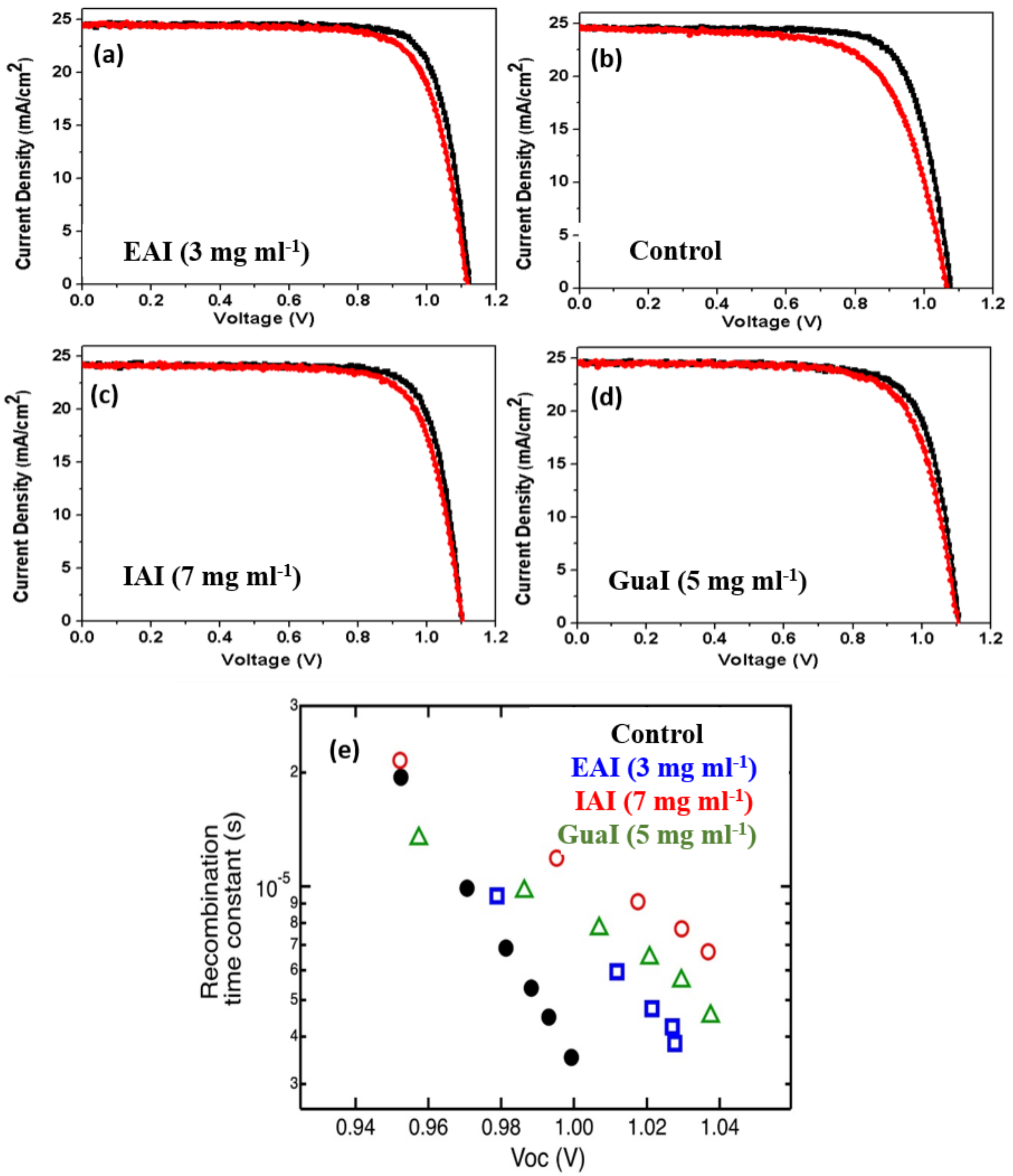

Supplementary Figure S6: Forward (black) and reverse scans (red) for a) control, and b) EAI, c) IAI, and d) GuaI modified devices with a scan rate of 10 mV/s, (e) Recombination time constants for various solar cells with and without surface treatments.

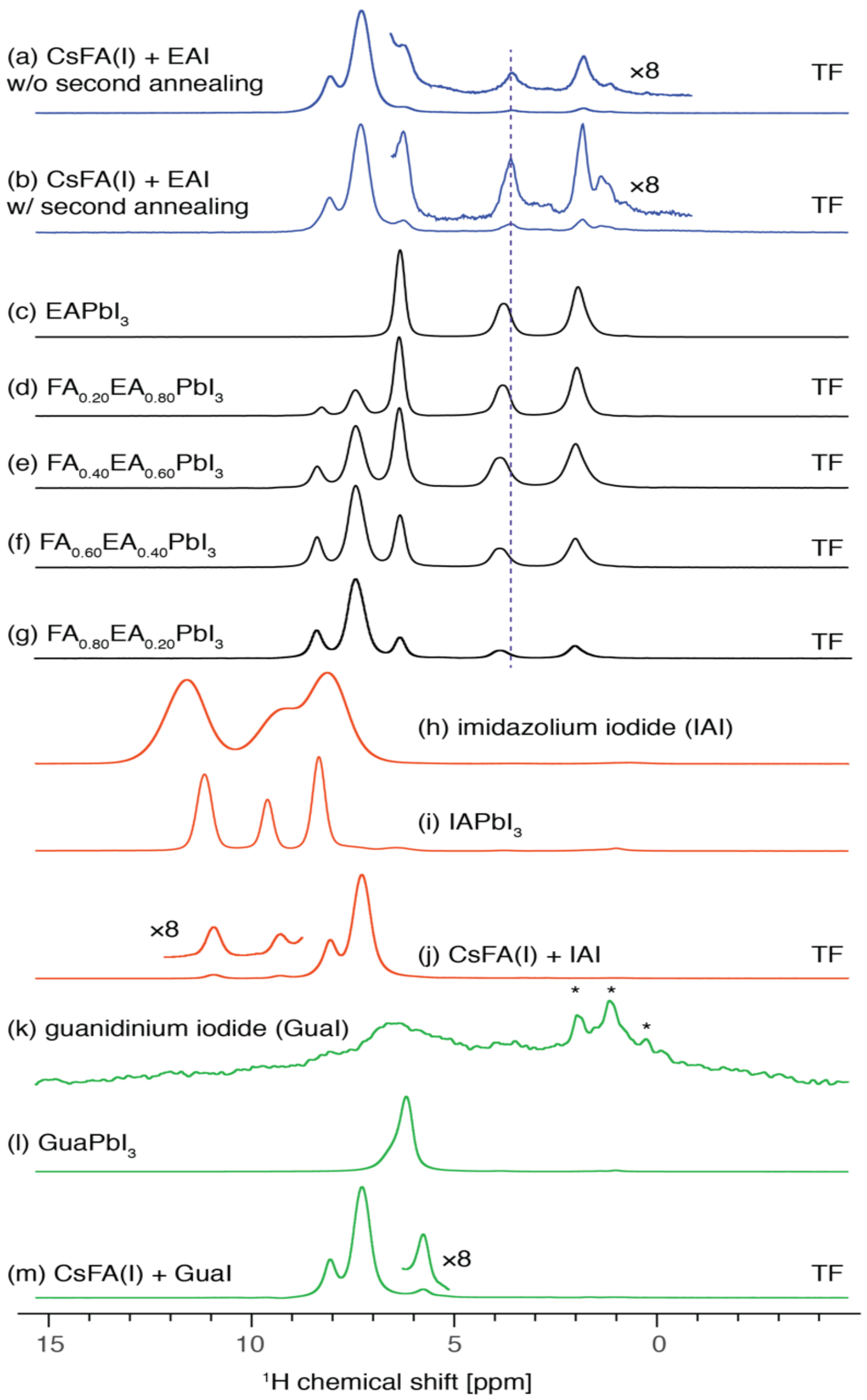

Supplementary Figure S7. ^{1}H solid-state MAS NMR spectra at 21.1 T, 298 K and 20 kHz MAS of bulk mechanochemical perovskite powders and thin films (indicated as "TF"). $FA_{0.93}Cs_{0.07}PbI_3$ thin film treated with 5 mg/ml EAI (a) without the second annealing step, (b) with the second annealing step. $FA_{1-x}EA_xPbI_3$ phases: (c) x=1.00 (bulk mechanochemical $EAPbI_3$), (d) x=0.80 (thin film), (e) x=0.60 (thin film), (f) x=0.40 (thin film), (g) x=0.20 (thin film), (h) neat imidazolium iodide, (i) imidazolium lead iodide, (j) $FA_{0.93}Cs_{0.07}PbI_3$ thin film treated with 7 mgml^{-1} IAI, (k) neat guanidinium iodide, (l) guanidinium lead iodide, (m) $FA_{0.93}Cs_{0.07}PbI_3$ thin film treated with 5 mgml^{-1} GuaI.

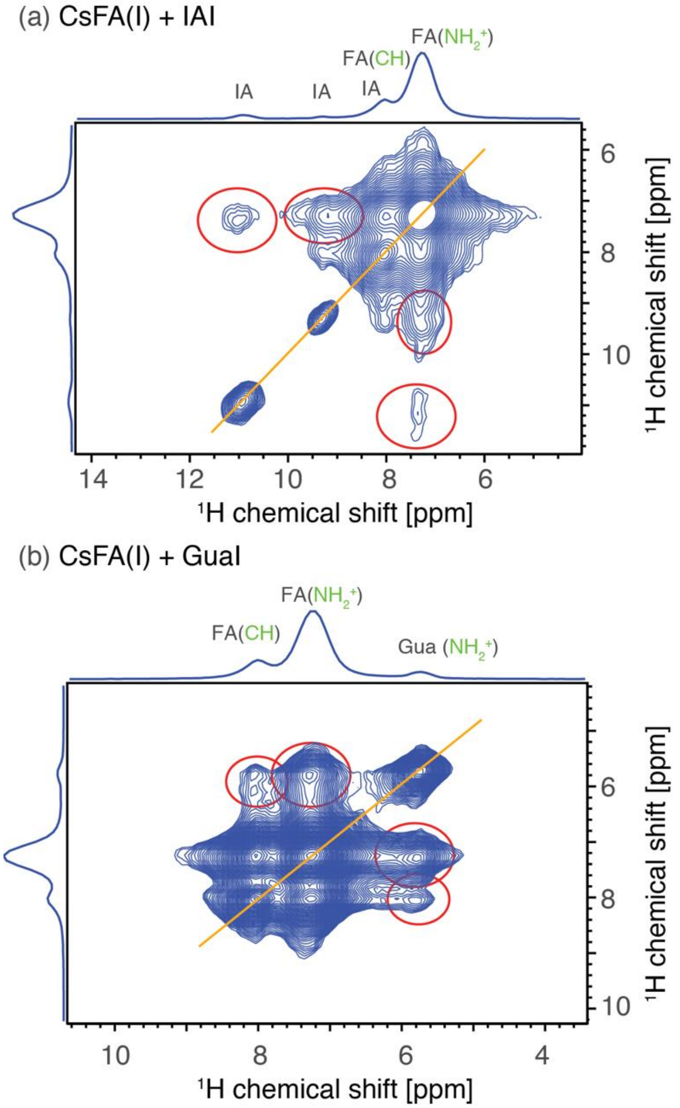

Supplementary Figure S8. ^{1}H solid-state MAS NMR spectra at 21.1 T, 298 K and 20 kHz MAS. ^{1}H-^{1}H spin diffusion experiments evidencing atomic-level proximity between FA, IAI and GuaI in a $FA_{0.93}Cs_{0.07}PbI_3$ thin film treated with (a) 7 mg/ml IAI (FA/IAI cross-peaks in red circles) and (b) 5 mgml^{-1} GuaI (FA/GuaI cross-peaks in red circles).

Supplementary Table S1. Summarized *J-V* characteristics of the best devices with varying EAI, IAI and GuaI contents in comparison to the control device:

	V_{oc} (V)	J_{sc} (mA/cm^2)	FF (%)	PCE (%)
**Control**	**1.08**	**24.50**	**76**	**20.52**
**Control/IPA**	**1.10**	**23.96**	**76**	**20.44**
**EAI**				
**3 mg ml^{-1}**	**1.12**	**24.14**	**81**	**22.40**
**5 mg ml^{-1}**	**1.15**	**24.60**	**77**	**22.23**
**7 mg ml^{-1}**	**1.15**	**23.50**	**68**	**18.75**
**10 mg ml^{-1}**	**1.17**	**23.00**	**63**	**17.30**
**IAI**				
**3 mg ml^{-1}**	**1.12**	**24.29**	**77**	**21.40**
**5 mg ml^{-1}**	**1.13**	**24.03**	**79**	**21.90**
**7 mg ml^{-1}**	**1.15**	**23.85**	**79**	**22.11**
**10 mg ml^{-1}**	**1.16**	**23.40**	**74**	**20.50**
**GuaI**				
**3 mg ml^{-1}**	**1.10**	**24.46**	**76**	**20.68**
**5 mg ml^{-1}**	**1.12**	**24.50**	**75**	**21.00**
**7 mg ml^{-1}**	**1.14**	**23.72**	**71**	**19.60**
**10 mg ml^{-1}**	**1.16**	**23.00**	**70**	**19.06**
**Mixed Cation**				
**EAI (mg ml^{-1})/IAI (7 mg ml^{-1})**				
	**1.14**	**21.97**	**0.71**	**18.1**
**EAI (3 mg ml^{-1})/GuaI (7 mg ml^{-1})**				
	**1.11**	**22.09**	**0.60**	**14.8**

Supplementary Table S2. Summarized J-V characteristics of the devices at forward and reverse scans at scan rate 10 mV/s^{-1} with varying EAI, IAI and GuaI contents in comparison to the control device:

	Scan dierction	J_{sc}(mA/Cm^2)	V_{oc} (V)	FF(%)	PCE(%)
**Control**	Forward	**24.32**	**1.063**	**70.3**	**18.54**
	Backward	**24.38**	**1.071**	**77.8**	**20.72**
**EAI (3 mg ml^{-1})**	Forward	**24.35**	**1.117**	**76.4**	**21.20**
	Backward	**24.36**	**1.123**	**80.4**	**22.30**
**IAI (7 mg ml^{-1})**	Forward	**24.23**	**1.102**	**75.2**	**20.50-**
	Backward	**24.14**	**1.103**	**79.4**	**21.60**
**GuaI (5 mg ml^{-1})**	Forward	**24.39**	**1.102**	**72.3**	**20.03**
	Backward	**24.45**	**1.106**	**75.3**	**20.90**

Supplementary Note 1: We have recently shown that solid-state magic angle spinning (MAS) NMR can be used to probe the atomic-level microstructure of multi-component lead halide perovskites [1] [2] [3] [4]. In particular, high-resolution ^{1}H NMR makes it possible to quantify the amount of organic cations in the final solid perovskite material. In order to establish whether or not annealing at 150 °C leads to evaporation of MA from the "CsMAFA" thin films, we have carried out ^{1}H solid-state MAS NMR measurements on the material prepared as a thin film (fig. S6). The reference ^{1}H spectrum of bulk mechanochemical $MAPbI_3$ exhibits two signals corresponding to the CH_3 (3.40 ppm) and NH_3^+ (6.38 ppm) groups of the MA cation (fig. 1a) while the "CsFA" material prepared as a thin film exhibits two signals corresponding to the CH (8.30 ppm) and NH_2^+ (7.53 ppm) groups of the FA cation. The "CsMAFA" composition exhibits two sets of signals, corresponding to FA (8.34 and 7.58 ppm) and MA (6.44 and 3.54 ppm). Quantification of the corresponding resonances yields 0.97 mol % FA and 0.03 mol % MA which is in perfect agreement with the stoichiometry of the precursor solution, indicating that MA is fully preserved in the final perovskite composition despite the relatively high annealing temperature

Supplementary Note 2: quantification of the passivation layer thickness.

The thickness of the 1D passivation layer can be quantified based on the quantitative ^{1}H MAS NMR spectrum of $(CH_5N_2)_{0.93}Cs_{0.07}PbI_3$ and simple geometrical considerations.

Perovskite layer thickness: 350 nm

Passivation layer thickness: 27 nm (by NMR), as detailed below.

Film dimensions: 2.5 cm x 1.75 cm (identical for both layers, this dimension will reduce in the final result)

Material density: 4.2 g/cm^3 (value for α-$FAPbI_3$)
Perovskite volume: 350 nm * 2.5 cm * 1.75 cm = 1.53125×10^{-7} dm^3
Perovskite mass: 0.643 mg
Perovskite molecular weight: 639 g/mol (where H: 4.7 g/mol)
Perovskite number of moles: 1.006 μmol (where H: μmol)

Integrals in the quantitative ^{1}H NMR spectrum of $(CH_5N_2)_{0.93}Cs_{0.07}PbI_3$ passivated with 5 mg/ml EAI:

$(CH_5N_2)_{0.93}Cs_{0.07}PbI_3$: 0.90 --- corresponding to --- 0.0074 μmol

EAPbI3 ($C_2H_8NPbI_3$): 0.10 --- corresponding to --- x mol

x = 0.00082 μmol of hydrogen in the 1D passivation layer.

$EAPbI_3$ molecular weight: 634 g/mol (where H: 8 g/mol)
Number of moles of EAPbI3 in the film: 0.00082 μmol/(8/634) = 0.06499 μmol
Mass of EAPbI3 in the film: 0.06499 μmol * 634 g/mol = 41.2 μg

Taking density of EAPbI3 as d=3.5 g/cm^3 (typical value for 1D haloplumbate phases, cf. e.g. APL Materials, **2018**, *6*, 114202, DOI: 10.1063/1.5046404) this yields the volume of the passivation layer: $1.17714286 \times 10^{-8}$ dm^3. Dividing by the film dimensions (2.5 cm * 1.75 cm = 0.0004375 m^2), **the passivation layer thicknes is: 27 nm**.

Supplementary Note 3: Description of Intensity Modulated Photovoltage Spectroscopy (IMVS)

Intensity Modulated Photovoltage Spectroscopy is an optoelectronic technique extremely useful in Solar cell technology to measure recombination time constants inside a device. The technique makes use of a potentiostat equipped with frequency response analyzer and an LED for stimulating the device under test (DUT). The potentiostat controls and monitors the LED and the Solar Cell, respectively.

The solar cell is at open circuit and is illuminated by the LED at a bias constant light. The LED is controlled in Galvanostatic mode at different bias currents. A small-amplitude sinusoidal current stimulus is added on top of the bias current sourcing the LED. The frequency of the sinusoidal stimulus is varied, thus probing the frequency response of the DUT for a defined spectral range.

The bias light defines the V_{oc} of the device and therefore the working point around which the small amplitude Voltage frequency response of the DUT is measured.

The transfer function is a complex function whose Imaginary and Real parts can be expressed as a function of frequency.

The Lifetime of the carriers τ inside the DUT is related to the frequency corresponding to the peak of the imaginary part by the following equation: $\tau=(2\pi f_{min})^{-1}$ [5].

Supplementary References

[1] D. J. Kubicki et al, Cation Dynamics in Mixed-Cation (MA) x(FA) 1– xPbI 3Hybrid Perovskites from Solid-State NMR, *J. Am. Chem. Soc.*, vol. 139, no. 29, pp. 10055–10061, Jul. 2017.

[2] D. J. Kubicki, D. Prochowicz, A. Hofstetter, S. M. Zakeeruddin, M. Gratzel, and L. Emsley, Phase Segregation in Cs-, Rb- and K-Doped Mixed-Cation (MA) x(FA) 1– xPbI 3Hybrid Perovskites from Solid-State NMR, *J. Am. Chem. Soc.*, vol. 139, no. 40, pp. 14173–14180, Sep. 2017.

[3] D. J. Kubicki et al, Formation of Stable Mixed Guanidinium–Methylammonium Phases with Exceptionally Long Carrier Lifetimes for High-Efficiency Lead Iodide-Based Perovskite Photovoltaics, *J. Am. Chem. Soc.*, vol. 140, no. 9, pp. 3345–3351, Feb. 2018.

[4] D. J. Kubicki, D. Prochowicz, A. Hofstetter, S. M. Zakeeruddin, M. Gratzel, and L. Emsley, Phase Segregation in Potassium-Doped Lead Halide Perovskites from 39K Solid-State NMR at 21.1 T, *J. Am. Chem. Soc.*, vol. 140, no. 23, pp. 7232–7238, May 2018.

[5] G. Schlichthorl et al, Band edge movement and recombination kinetics in dye-sensitized nanocrystalline TiO2 solar cells: A study by intensity modulated Photovoltage Spectroscopy, J. Phys. Chem. B 1997, 101, 8141- 8155.
